# Machine Learning in the Management of Patients Undergoing Catheter Ablation for Atrial Fibrillation: Scoping Review

**DOI:** 10.2196/60888

**Published:** 2025-02-10

**Authors:** Aijing Luo, Wei Chen, Hongtao Zhu, Wenzhao Xie, Xi Chen, Zhenjiang Liu, Zirui Xin

**Affiliations:** 1 The Second Xiangya Hospital Central South University Changsha China; 2 School of Life Sciences Central South University Changsha China; 3 Key Laboratory of Medical Information Research (Central South University) College of Hunan Province Changsha China; 4 Clinical Research Center For Cardiovascular Intelligent Healthcare In Hunan Province Changsha China; 5 Information and Network Center The Second Xiangya Hospital Central South University Changsha China; 6 The Third Xiangya Hospital Central South University Changsha China; 7 Department of Cardiology The Second Xiangya Hospital Central South University Changsha China

**Keywords:** atrial fibrillation, catheter ablation, deep learning, patient management, prognosis, quality assessment tools, cardiac arrhythmia, public health, quality of life, severe medical condition, electrocardiogram, electronic health record, morbidity, mortality, thromboembolism, clinical intervention

## Abstract

**Background:**

Although catheter ablation (CA) is currently the most effective clinical treatment for atrial fibrillation, its variable therapeutic effects among different patients present numerous problems. Machine learning (ML) shows promising potential in optimizing the management and clinical outcomes of patients undergoing atrial fibrillation CA (AFCA).

**Objective:**

This scoping review aimed to evaluate the current scientific evidence on the application of ML for managing patients undergoing AFCA, compare the performance of various models across specific clinical tasks within AFCA, and summarize the strengths and limitations of ML in this field.

**Methods:**

Adhering to the PRISMA-ScR (Preferred Reporting Items for Systematic Reviews and Meta-Analyses extension for Scoping Reviews) guidelines, relevant studies published up to October 7, 2023, were searched from PubMed, Web of Science, Embase, the Cochrane Library, and ScienceDirect. The final included studies were confirmed based on inclusion and exclusion criteria and manual review. The PROBAST (Prediction model Risk Of Bias Assessment Tool) and QUADAS-2 (Quality Assessment of Diagnostic Accuracy Studies-2) methodological quality assessment tools were used to review the included studies, and narrative data synthesis was performed on the modeled results provided by these studies.

**Results:**

The analysis of 23 included studies showcased the contributions of ML in identifying potential ablation targets, improving ablation strategies, and predicting patient prognosis. The patient data used in these studies comprised demographics, clinical characteristics, various types of imaging (9/23, 39%), and electrophysiological signals (7/23, 30%). In terms of model type, deep learning, represented by convolutional neural networks, was most frequently applied (14/23, 61%). Compared with traditional clinical scoring models or human clinicians, the model performance reported in the included studies was generally satisfactory, but most models (14/23, 61%) showed a high risk of bias due to lack of external validation.

**Conclusions:**

Our evidence-based findings suggest that ML is a promising tool for improving the effectiveness and efficiency of managing patients undergoing AFCA. While guiding data preparation and model selection for future studies, this review highlights the need to address prevalent limitations, including lack of external validation, and to further explore model generalization and interpretability.

## Introduction

### Background

Atrial fibrillation (AF) is the most common type of cardiac arrhythmia [1], and its incidence has risen steadily over the past 3 decades, making it an urgent public health problem worldwide [2,3]. While AF substantially impacts patients’ quality of life, it also increases the risk of serious medical conditions [4-7], such as stroke and thromboembolism. This contributes to long-term rises in both morbidity and mortality rates [8] as well as a growing strain on health care costs [9].

Catheter ablation (CA) represents the most effective clinical intervention currently available for AF [10]. When patients receive their first CA treatment, the effectiveness rate can reach 60%-80% within 1 year [11,12]. However, the long-term efficacy is not optimal , and the success rate decreases to 50%-60% at 3 to 5 years [13-15]. The type and degree of AF, cardiac stroma, obesity, alcohol consumption, and obstructive sleep apnea syndrome are all known to be potential contributors to high rates of recurrence in the long term [16,17]. In addition, the surgical modality and ablation strategy used in treatment have an impact, particularly for those patients who require additional non–pulmonary vein (PV) ablations [18]. Consequently, developing tailored management strategies that consider different patient conditions, optimizing the surgical approach and enhancing its long-term efficacy, is a challenge that needs to be resolved in this field.

In clinical practice, to better manage patients, clinical risk scores (such as APPLE [19]) specifically for predicting postoperative recurrence of AF CA (AFCA) have emerged in the past decade, or attempts have been made to use other scores (such as CHADS_2_ [20] and CHA_2_DS_2_-VASc [21]) to evaluate patients’ postoperative rhythm outcomes after AFCA [22]. These risk scores typically use a limited number of risk factors as predictors. Although this simplicity facilitates their adoption and use in clinical settings, it concurrently caps their predictive effectiveness. In addition, these scoring models predominantly rely on the evaluation of specific biomarkers in previous literature when selecting predictors. This approach may easily lead to the neglect of some potential predictors [23], thus failing to truly capture the complexity of the AFCA recurrence mechanism and making it difficult to truly meet the pursuit of efficient and personalized patient management.

Rapidly developing machine learning (ML) techniques hold significant promise as a formidable support in grappling with the complexities of this challenge. In recent years, the ability of ML to analyze large biomedical datasets, such as electronic health records (EHRs), medical imaging, and multiomics data, has made it increasingly popular in medical research [24,25]. ML models have become promising tools for enhancing the precision and efficiency of AF diagnosis and treatment. By analyzing complex, multidimensional datasets, they help identify individuals at high risk for AF, uncover risk factors and biomarkers associated with the disease’s progression, and guide personalized interventions for prognosis. One branch of ML, deep learning (DL), stands out due to its powerful capabilities in automatic feature recognition and prediction. In processing complex data types such as images and electrophysiological signals, DL has demonstrated performance comparable to that of human experts [26,27]. Several studies have attempted to apply ML technology to the management of patients undergoing AFCA. As the application prospects of ML technology in this field become increasingly clear, a comprehensive overview and evaluation of the existing relevant literature is needed.

### Objectives

The purpose of this scoping review is to systematically review the current progress in using ML technology in the management of patients undergoing AFCA. Specifically, the studies discussed in this review explore a wide range of ML models and multiple data modalities, including EHR, imaging omics, and cardiac electrophysiological signals. We will summarize how these studies prepare data, select models, and evaluate results across different clinical tasks. In addition, we will emphasize the advantages and limitations of ML technology in this field based on the integration and comparison of these study findings.

## Methods

This scoping review was conducted following the PRISMA-ScR (Preferred Reporting Items for Systematic reviews and Meta-Analyses extension for Scoping Reviews) guidelines [28].

### Information Sources and Search Strategies

A comprehensive search was conducted to identify relevant studies across 5 databases as of October 7, 2023: PubMed, Web of Science, Embase, the Cochrane Library, and ScienceDirect. The specific search strategies used for each database are detailed in Table S1 in Multimedia Appendix 1. In addition to obtaining literature through search formula, additional relevant articles (3/246, 1.2%) were manually identified by using a forward-backward snowballing approach.

### Study Inclusion and Exclusion Criteria

In line with the thematic focus and objectives of our review, the studies included in this scoping review were required to meet the eligibility criteria outlined in Textbox 1. Studies were excluded from this scoping review if they met any of the exclusion criteria.

**Inclusion and exclusion criteria for published studies applying machine learning (ML) technology for the management of patients undergoing catheter ablation for atrial fibrillation.**

**Inclusion criteria**

- Article type: articles (peer reviewed and formally published)
- Study topic: specializes in the management of patients undergoing catheter ablation for atrial fibrillation
- Study participants: real patients with atrial fibrillation
- Study design: ML techniques were clearly applied and described; results of the study and the ML model performance outcome metrics were explicitly presented
- Language of publication: English

**Exclusion criteria**

-
  Article type: reviews; meta-analyses; editorials; case reports; editorial materials; other types of publications
- Study topic: atrial fibrillation disease detection; risk prediction of new onset of atrial fibrillation
- Study participants: only used fictional patient data
- Study design: did not apply ML techniques; did not explicitly report on the model outcome metrics
- Language of publication: non-English

### Study Selection

After removing duplicates, all retrieved studies were manually screened by title and abstract to exclude those clearly not aligned with the objectives of this review. Studies that passed this initial screening were then downloaded in full and assessed based on the inclusion and exclusion criteria during a full-text review. This process was independently conducted by 2 reviewers, with any disagreements resolved through consultation with a third reviewer to reach a consensus.

### Data Extraction

Two reviewers independently extracted data from the selected studies, and a third reviewer was responsible for assessing and addressing any discrepancies and biases in data extraction. The following data items were extracted: author names and publication years, data sources, dataset sizes, ML models, model variables, model tasks (such as prediction or detection), and model results. If multiple ML models were used in a study, we will report only the one with the best performance.

### Quality Assessment

We selected the widely recognized Quality Assessment of Diagnostic Accuracy Studies-2 (QUADAS-2) [29] and Prediction model Risk Of Bias Assessment Tool (PROBAST) [30] as methodological quality assessment tools. We divided the included studies into 2 subgroups for independent evaluation based on different research objectives, namely, subgroup A: studies that aim at image classification or segmentation, assessed using an adapted version of QUADAS-2; and subgroup B: with the goal of predicting patient prognosis, assessed using PROBAST. This assessment was independently conducted by 2 reviewers, and any disagreements or discrepancies that arise during the assessment process were resolved with the involvement of a third reviewer.

During our review, we adjusted some signal questions in QUADAS-2 as needed. Tables S2 and S3 in Multimedia Appendix 1 report the specific signal questions and assessment instructions for the tools used in the quality assessment process for this review.

## Results

### Study Selection and Study Characterization

**Figure 1:**
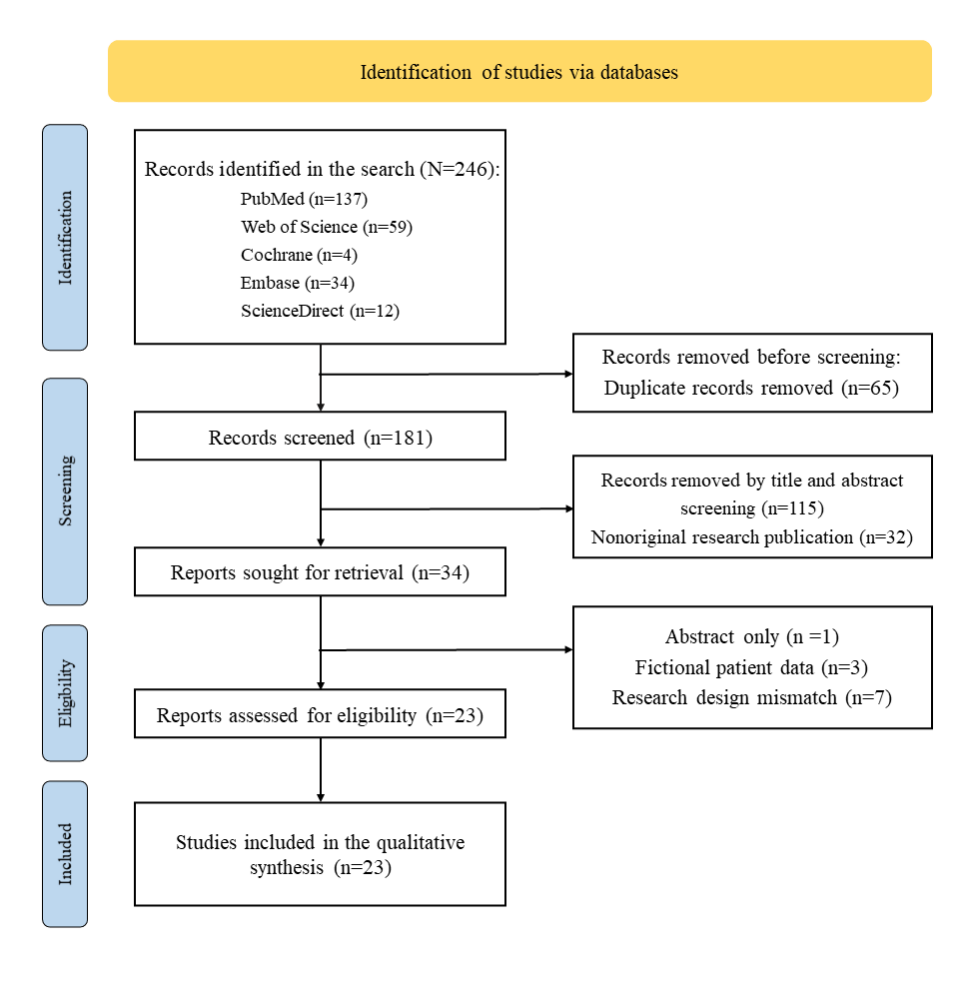
PRISMA (Preferred Reporting Items for Systematic Reviews and Meta-Analyses) flowchart of study selection.

A total of 246 studies were identified from different databases using the method described in the Information Sources and Search Strategies section (Figure 1). After excluding duplicate publications (65/246, 26.4%), nonarticle publications (32/246, 13%), and studies that did not match the research objectives of this scoping review (115/246, 46.7%), 34 (13.8%) studies remained for full-text assessment. Ultimately, 23 studies were deemed eligible and were included for data extraction and synthesis. Table S4 in Multimedia Appendix 1 provides a detailed list of the studies excluded after full-text assessment, along with the reasons for their exclusion.

Table 1 provides the basic characteristics of the studies included in this review. The publication of these studies was concentrated within the past 5 years, which can be attributed to the maturation of ML technologies and the growing interest in precision medicine among researchers in the field of AF. The included studies were conducted in 9 different countries, with the United States (7/23, 30%) having the largest number of studies, followed by China (5/23, 22%) and the United Kingdom (4/23, 17%). This diversity in publication countries reflects the global interest in using ML models for the management of patients undergoing AFCA. Regarding the types of models used, most of the included studies (14/23, 61%) used DL techniques.

**Table 1:** Basic characteristics of the included studies (N=23).

Characteristic		Studies, n (%)
**Country of study**		
	United States	7 (30)
	China	5 (22)
	United Kingdom	4 (17)
	Korea	2 (9)
	Spain	1 (4)
	The Netherlands	1 (4)
	Canada	1 (4)
	Italy	1 (4)
	Japan	1 (4)
**Year of publication**		
	2023	3 (13)
	2022	7 (30)
	2021	6 (26)
	2020	6 (26)
	2019	1 (4)
**Type of models**		
	Machine learning	23 (100)
	Deep learning	14 (61)
**Key medical tasks**		
	Patient prognosis prediction	16 (70)
	Ablation targets identification	3 (13)
	Ablation strategy improvement	4 (17)

Regarding the application direction of ML in AFCA patient management, this review divides the included studies into 3 subgroups corresponding to 3 key medical tasks based on the purpose of the model. The first and most common focus is the development of prognostic models for patients undergoing AFCA (16/23, 70%). These studies aim to provide accurate, timely, and personalized postoperative risk predictions for patients undergoing AFCA [31-46]. Although these studies share similar objectives, they use diverse types of data, with EHRs being the primary data source. The second key task focuses on the identification of ablation targets (3/23, 13%), aiming to use the feature extraction capabilities of DL models to identify potential ablation targets, such as rotors [47,48] and focal sources [49], from electrophysiological and medical imaging data. Third, some studies focus on using preoperative imaging data of patients to improve ablation strategies (4/23,17%), assisting physicians in recognizing interpatient differences to guide ablation treatments [50-53].

### Characteristics of the Data in the Included Studies

When developing ML models for patient management, key factors to consider include dataset size, data modality, and resource availability. Table 2 outlines the wide range of data modalities covered in this review, and we note that there is a wide variation in the choice of variables and the number of features across studies, which highlights the diversity and complexity of factors associated with influencing patient management in AFCA.

**Table 2:** Summary of data use of the included studies.

Study	Patient cohort		Input data for the model	Data sources
	Patients, n	Time frame		
Liu et al [50], 2020	358	October 2004 to December 2017	PVCT^a^	Taipei Veterans General Hospital
Firouznia et al [31], 2021	203	2013 to 2016	CT^b^, age, LAV^c^, AF^d^ type, and catheter ablation technique	Cleveland Clinic
Atta-Fosu et al [32], 2021	68	July 2015 to November 2016	CT, age, sex, LAV, LVEF^e^, BMI, sinus rhythm at the time of ablation, AF type, and catheter ablation technique	Cleveland Clinic
Muizniece et al [51], 2021	122	2011 to 2016	LGE-MRI^f^	2018 STACOM Split Challenge and St. Thomas Hospital
Muffoletto et al [52], 2021	122	2011 to 2016	LGE-MRI	2018 STACOM Split Challenge and St. Thomas Hospital
Shade et al [33], 2020	32	December 2011 to December 2015	Personalized atrial computational modeling and LGE-MRI	Hopkins University Hospital
Roney et al [34], 2022	100	NR^g^	Different types of fibrosis in the atrial matrix, fibrograms, AF induction protocols, ERP^h^ values, PVI^i^ size, visual fibrosis score, total surface area of left atrium, total surface area of the PVs, fibrotic area of PVs, LVEF, BMI, age, female, congestive HF^j^, hypertension, diabetes mellitus, stroke, coronary artery disease, and type of AF	St. Thomas Hospital
Yang et al [53], 2020	190	2011 to 2018	LGE-MRI	Local regional ethics committee
Hwang et al [35], 2020	606	July 2008 to July 2019	Transthoracic echocardiography	Chang Gung Memorial Hospital
Liao et al [49], 2021	78	NR	uEGM^k^	Focal Source and Trigger randomized controlled trial (Chauhan et al [54])
Ríos-Muñoz et al [47], 2022	48	NR	uEGM, bEGM^l^, and uLAT^m^	Gregorio Marañón University General Hospital
Alhusseini et al [48], 2020	35	Up to December 2017	EGM^n^	Comparison of Algorithms for Rotational Evaluation in Atrial Fibrillation Registry
An et al [36], 2022	12	January 2015 to January 2019	3D atrial endocardial unipolar and bipolar voltage map	the Dallas VA Medical Center
Tang et al [37], 2022	156	2015 to 2017	EGM, ECG^o^, age, gender, height, weight, BMI, ethnicity and race, hypertension, hyperlipidemia, TIA^p^, stroke, CAD^q^, diabetes mellitus, chronic kidney disease, congestive HF, OSA^r^, type of AF, previous history of AF ablation, LVEF, LAD^s^, LAV, LASA^t^, and LASI^u^	Stanford University Tertiary Referral Center
Li et al [38], 2019	14	NR	BSPM^v^	West China Hospital of Sichuan University
Saiz-Vivo et al [39], 2021	74	October 2005 to July 2014	Clinical data and heart rate variability characteristics extracted from ICM^w^	Reveal LINQ Usability Study and clinical data from a single center
Jiang et al [40], 2023	1618	January 2012 to May 2019	12-lead ECG	Guangdong Provincial People’s Hospital
Lee et al [41], 2022	177	February 2017 to October 2020	Age, sex, height, weight, hypertension, AF type, AF duration, LAD, LAMI^x^, LVEF, and eGFR^y^	Chungbuk National University Hospital
Park et al [42], 2022	1872	2009 to 2018	Age, female, AF type, BMI, heart failure, hypertension, diabetes mellitus, stroke or TIA, vascular disease, left atrium dimension, LVEF, EEM^z^, creatinine, hemoglobin, and preablation PR interval	Yonsei AF Ablation Cohort Database
Hung et al [43], 2020	11,334	January 2013 to December 2013	Age, total hospital discharges, number of diagnoses, number of chronic diseases, length of hospitalization, number of surgeries, gender, diabetes, hypertension, hypothyroidism, COPD^aa^, renal failure, depression, peripheral vascular disease, obesity, size of hospital beds, type of hospital, and discharge status	National Readmission Database 2013
Saglietto et al [44], 2023	3128	April 2012 to April 2015	LVEDV^ab^, estimated glomerular filtration rate, BMI, age, pre- and post–left atrium diameters, LVEF, CHAD2 DS2-VASc score, dyslipidemia, AFL^ac^, type of ablation procedure, type of AF, structural heart disease, hypertension, baseline sinus rhythm, gender, abnormal ECG, heart failure, CAD, and smoking history	European Society of Cardiology-European Heart Rhythm Association Long-Term Registry for Atrial Fibrillation Ablation
Ma et al [45], 2023	471	January 2018 to December 2020	Age, male, BMI, diastolic blood pressure, systolic blood pressure, smoking history, CHA2DS2-VASc, HABLED score, AF duration, hypertension, CAD, type 2 diabetes mellitus, chronic HF, atrial septal defect, LAD, LVEF, and white blood cell counts	The First Affiliated Hospital of Air Force Medical University
Zhou et al [46], 2022	310	Jun 2016 - Oct 2019	NT-proBNP^ad^, paroxysmal AF, LAAV^ae^, and LAV	Toyo University Ohashi Medical Center

^a^PVCT: pulmonary vein computed tomography.

^b^CT: computed tomography.

^c^LAV: left atrial volume.

^d^AF: atrial fibrillation.

^e^LVEF: left ventricular ejection fraction.

^f^LGE-MRI: late gadolinium-enhanced cardiac magnetic resonance.

^g^NR: not reported.

^h^ERP: effective refractory period.

^i^PVI: pulmonary vein isolation.

^j^HF: heart failure.

^k^uEGM: unipolar endocardial electrogram.

^l^bEGM: bipolar endocardial electrogram.

^m^uLAT: unipolar localized activation time.

^n^EGM: electrogram.

^o^ECG: electrocardiogram.

^p^TIA: transient ischemic attack.

^q^CAD: coronary artery disease.

^r^OSA: obstructive sleep apnea.

^s^LAD: left atrial diameter.

^t^LASA: left atrial surface area.

^u^LASI: left atrial spherical index.

^v^BSPM: body surface potential map.

^w^ICM: implantable cardiac monitor.

^x^LAMI: left atrial mass index.

^y^eGFR. estimated glomerular filtration rate.

^z^EEM: peak transmitral flow velocity (E), and tissue Doppler echocardiography of the peak septal mitral annular velocity (em).

^aa^COPD: chronic obstructive pulmonary disease.

^ab^LVEDV: left ventricular end-diastolic volume.

^ac^AFL: atrial flutter.

^ad^NT-proBNP: N-terminal pro-brain natriuretic peptide.

^ae^LAAV: left atrial appendage volume.

The characteristics of the data used in the included studies are provided in Table 3, which summarizes dataset size, data modality, data source, and whether the data were multicenter. There was substantial variation among the included studies in the size of the dataset used to develop the ML model, with patient numbers ranging from 8 to 5872, but few studies adequately described their methodology or reasons for determining the sample size, in contrast to studies of randomized controlled trials based on statistical methods. In terms of data resource availability, only 17% (4/23) of the studies used publicly available datasets of patients with AF (2013 Nationwide Readmissions Database [43], 2018 Atria Segmentation Data [51,52], and European Society of Cardiology-European Heart Rhythm Association Long-Term Registry for AF Ablation [44]) for model development or validation, while most of the remaining studies (19/23, 83%) used private single-center retrospective datasets. In addition, 17% (4/23) of the studies used multicenter datasets in model development or external datasets for validation after model development [39,44,51,52].

**Table 3:** Characteristics of the data used in the included studies (N=23).

Characteristics of used data		Studies, n (%)	References
**Size of dataset**			
	<50	5 (22)	[33,36,38,47,48]
	50-100	4 (17)	[32,34,39,49]
	101-200	5 (22)	[37,41,51-53]
	201-500	4 (17)	[31,45,46,50]
	501-1000	1 (4)	[35]
	>1000	4 (17)	[40,42-44]
**Data modality**			
	Electronic health records	10 (43)	[31,32,34,37,41-46]
	Medical imaging	9 (39)	[31-35,50-53]
	Electrophysiological signal	7 (30)	[36-38,40,47-49]
	Biomedical simulation	2 (9)	[33,34]
	Other	1 (4)	[39]
	Multimodal input used	4 (17)	[31,32,34,37]
**Availability of data source**			
	Private	19 (83)	[31-42,45-50,53]
	Public	4 (17)	[43,44,51,52]
**Multicenter data used**			
	Yes	4 (17)	[39,44,51,52]
	No	19 (83)	[31-38,40-43,45-50,53]

**Figure 2:**
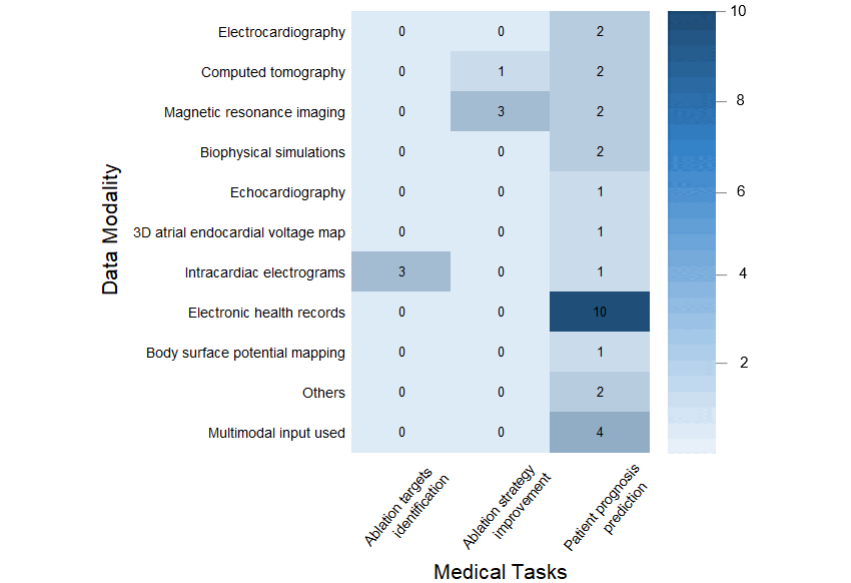
Evidence heat map of data modality by machine learning model (grouped by medical task).

To provide a more intuitive integration and comparison of the data modalities of the included studies, an evidence heat map was used to visualize the results (Figure 2), which highlights and reveals the medical imaging data and electrophysiological signal data involved in the included studies. In studies aimed at predicting patient prognosis, the most commonly used data type was the EHR (10/16, 63%), and it is worth noting that a small number of studies (4/16, 25%) in this segment also used multimodal data, combining either imaging data or electrocardiographic signals (or both) with clinical features to build model, and reported superior model performance relative to their constructed unimodal models. Intracardiac electrograms (EGMs) are a key data modality in studies focused on identifying ablation targets, whereas imaging data are commonly used in studies aimed at improving ablation strategies.

### Modeling Approaches and Performance

Distinct medical tasks and research objectives necessitate the application of various ML techniques. Table 4 provides the ML models constructed in each study and the specific values of their performance indicators. It should be noted that if >1 ML model was constructed in 1 study at the same time, only the optimal model performance among them will be reported in this review, and the complete information of the ML models is provided in Table S2 in Multimedia Appendix 1. In this review, if a study reports multiple ML models simultaneously, we will report only the optimal model performance among them. Table 4 provides details on the used ML techniques, medical tasks, and model performances in the included studies.

The classical ML models covered in this review are diverse, with random forest (6/23, 26%) and support vector machines (6/23, 26%) appearing most frequently, and in some studies, although they are not the best-performing models, they have been applied for feature selection or as comparative models. Of the 23 studies, 5 (22%) used the k-nearest neighbors algorithm, 3 (13%) used logistic regression, and 2 (9%) used the decision tree. In addition, 17% (4/23) of the studies used integrated learning models such as XGBoost and AdaBoost. It is important to note that DL models represented by convolutional neural network (CNN) and RestNet were the most frequently used as performance-optimized models in all the included studies, particularly in those studies that used image data and electrical signal data as model inputs, and 61% (14/23) of the studies reported performance-optimized models belonging to the DL type. Specifically, CNN and its variants were applied in 30% (7/23) of the studies, and ResNet was applied in 9% (2/23) of the studies.

In terms of model performance evaluation indicators, the main ones used were area under the receiver operating characteristic curve (AUC; 17/23, 74%), sensitivity and specificity (11/23, 48%), and accuracy (11/23, 48%), while some studies chose to assess the model performance with metrics such as *F*_1_-score. From the information in Tables 4 and 5, it can be seen that the performance of ML models in the included studies was good overall. Among them, 74% (17/23) of the studies reported the AUC values of the ML models, and the values of this performance indicator ranged from 0.5 to 0.98. The average AUC for ML models predicting patient prognosis was 0.8 (SD=0.11), with a median of 0.83 (IQR 0.74-0.86), and 13% (3/23) of the studies had a model AUC >0.90. Furthermore, 48% (11/23) of the studies reported model sensitivity and specificity, with sensitivity ranging between 0.71 and 0.97 and specificity ranging from 0.79 to 0.99. In terms of accuracy, 48% (11/23) of the studies reported this statistic, with values ranging from 0.72 to 0.99. All models aimed at improving ablation strategies reported accuracy, with an average of 0.87.

**Table 4:** Performance for each machine learning model according to the medical tasks (N=23).

Medical tasks and proposed model		AUC^a^	Sensitivity	Specificity	Accuracy	Other
**Patient prognosis** **prediction**						
	RF^b^ [31]	0.87	NR^c^	NR	NR	NR
	RF [42]	0.965	0.96	0.89	NR	NR
	RF [44]	0.721	NR	NR	NR	NR
	RF [45]	0.667	NR	NR	NR	NR
	XGboost [32]	0.78	NR	NR	NR	NR
	QDA^d^ [33]	0.82	0.82	0.89	NR	NR
	SVM^e^ [34]	0.85	NR	NR	NR	*F*_1_-score=0.8
	CNN^f^ [35]	0.861	0.803	0.789	0.796	NR
	CNN [37]	0.859	NR	NR	NR	NR
	CNN-SVM [38]	NR	0.88	0.96	0.96	NR
	CNN [40]	0.84	0.723	0.95	0.92	*F*_1_-score=0.71
	CNN [46]	0.76	NR	NR	NR	CI^g^=0.76
	KNN^h^ [43]	0.91	0.713	0.991	0.854	PR=0.886
	OWV^i^ [39]	NR	0.76	0.87	0.82	NR
	MLP^j^ [36]	0.5	NR	NR	0.875	*F*_1_-score=0.933
	MLP [41]	0.766	NR	NR	NR	NR
**Ablation targets identification**						
	ResNet [49]	0.980	0.781	0.822	NR	NR
	CRNN^k^ [47]	0.81	NR	NR	NR	MCC^l^=0.680
	CNN [48]	NR	0.97	0.93	0.95	NR
**Ablation strategy improvement**						
	ResNet [50]	0.88	0.75	0.957	0.886	NR
	Reinforcement Q-learning [51]	NR	NR	NR	0.72	NR
	MVVT^m^ [53]	NR	0.92	0.99	0.99	DI^n^=93.11
	CNN [52]	NR	NR	NR	0.865	NR

^a^AUC: area under the curve.

^b^RF: random forest.

^c^NR: not reported.

^d^QDA: quadratic discriminant analysis.

^e^SVM: support vector machine.

^f^CNN: convolutional neural network.

^g^CI: C-index.

^h^KNN: K-nearest neighbor.

^i^OWV: optimal weighted voting.

^j^MLP: multilayer perceptron.

^k^CRNN: convolutional recurrent neural network.

^l^MCC: Matthews correlation coefficient.

^m^MVTT: fully automatic multiview dual-task recursive attention model.

^n^DI: Dice score.

**Table 5:** Statistical validation of the machine learning models.

Statistics			Studies, n (%)		Medical tasks involved (fraction) and corresponding study reference
**AUC (%^a^)**					
	<70	2 (9)		Patient prognosis prediction (2/2): [36,45]	
	70-79	4 (17)		Patient prognosis prediction (4/4): [32,41,44,46]	
	80-85	4 (17)		Patient prognosis prediction (3/4): [33,34,40] Ablation targets identification (1/4): [47]	
	86-90	4 (17)		Patient prognosis prediction (3/4): [32,35,37] Ablation strategy improvement (1/4): [50]	
	>90	3 (13)		Patient prognosis prediction (2/3): [42,43] Ablation targets identification (1/3): [49]	
**Sensitivity (%^b^)**					
	<85	7 (30)		Patient prognosis prediction (5/7): [33,35,39,40,43] Ablation targets identification (1/7): [49] Ablation strategy improvement (1/7): [50]	
	85-89	1 (4)		Patient prognosis prediction (1/1): [38]	
	90-95	1 (4)		Ablation strategy improvement(1/1): [53]	
	>95	2 (9)		Patient prognosis prediction (1/2): [42] Ablation targets identification (1/2): [48]	
**Specificity (%^c^)**					
	<85	2 (9)		Patient prognosis prediction (1/2): [35] Ablation targets identification (1/2): [49]	
	85-89	3 (13)		Patient prognosis prediction (3/3): [33,39,42]	
	90-95	2 (9)		Patient prognosis prediction (1/2): [40] Ablation targets identification (1/2): [48]	
	>95	4 (17)		Patient prognosis prediction (2/4): [38,43] Ablation strategy improvement (2/4): [50,53]	
**Accuracy (%^d^)**					
	<80	2 (9)		Patient prognosis prediction (1/2): [35] Ablation strategy improvement (1/2): [51]	
	80-86	2 (9)		Patient prognosis prediction (2/2): [39,43]	
	87-92	4 (17)		Patient prognosis prediction (2/4): [36,40] Ablation strategy improvement (2/4): [50,52]	
	>92	3 (13)		Patient prognosis prediction (1/3): [38] Ablation targets identification (1/3): [48] Ablation strategy improvement (1/3): [53]	

^a^AUC: area under the curve; AUC values were mentioned in 17 included studies.

^b^Sensitivity was mentioned in 11 included studies.

^c^Specificity was mentioned in 11 included studies.

^d^Accuracy was mentioned in 11 included studies.

### Findings of the Included Studies

#### Identifying AFCA Targets

Three studies discussed the application of ML techniques in identifying potential targets for CA in AF [47-49]. Clinically, electrophysiologists must resort to time-consuming and complex invasive cardiac electrophysiological examinations to confirm ablation targets; deep neural networks can help improve this process. Liao et al [49] applied ResNet to classify raw intracardiac EGMs during AF, accurately identifying patients’ putative focal source target site with performance approximating that of human experts, obtaining an AUC of 0.98. Two other studies [47,48] applied CNN to detecting rotors in the intracardiac EGMs, with one of the them taking into account the interpretability of DL model and using gradient-weighted class-activation mapping (Grad-CAM) to explore how the model classifies AF electrical patterns [48], and the other designed the convolutional recurrent neural network from the perspective of improving model, while experimenting with multiple types of input signals, resulting in improved classification performance of the model.

#### Improving CA Strategies for AF

Due to varying degrees of individualization among patients, the development of a rational and effective strategy for AFCA relies on the judgment and surgical experience of the physician [16]. Notably, 17% (4/23) of the studies demonstrated the potential of DL to improve this process [50-53]. A common factor among these studies is that they were all based on imaging data. Liu et al [50] used the patient’s preablation PV computed tomography (CT) images to construct a ResNet-based prediction model, which identified potential non-PV triggering factors before ablation treatment with an AUC of 0.88, thereby prompting physicians to perform necessary additional ablation on the basis of PV isolation. In 3 other studies, cardiac late gadolinium-enhanced cardiac magnetic resonance (LGE-MRI) was used [51-53]. Muffoletto et al [52] and Muizniece et al [51] used CNN and reinforcement learning (Q-Learning) algorithms, respectively, to directly engage in the process of formulating personalized ablation strategies for patients with AF and achieved success rates of 79% and 72%, respectively, in their respective test sets. Yang et al [53] proposed a joint segmentation method based on the multiview two-task recursive attention model to provide clinicians with segmented left ventricular (LV) anatomical structures and LV scars directly and simultaneously from LGE-MRI, which can be used to provide valuable guidance for ablation treatment.

#### Prognosis for Patients With AFCA

The high long-term recurrence rate of CA has been troubling patients and clinicians. Of the 23 included studies, 16 (70%) focused on the prognosis of patients after AFCA. Furthermore, 35% (8/23) of the studies [38,40-46] attempted to predict the risk of postprocedural recurrence of AF using easily accessible preprocedural patient clinical characteristics, with commonly used variables including age, gender, BMI, comorbidities, and electrocardiogram (ECG), but their modeling performance varied widely, with reported AUC ranging from 0.667 to 0.965. Four studies [31,32,34,37] chose to fuse features from multiple modalities as the input of ML model for recurrence prediction of AF. For example, Tang et al [37] proposed a CNN-based multimodal fusion framework to predict AF recurrence 1 year after CA using patients’ clinical features, EGMs, ECG, and their combinations. The comparison of multiple experimental results showed that the recurrence prediction AUC of the multimodal feature fusion model was improved by 13.2% (0.86 vs 0.76) compared with the single-modality model. Independent prediction of AF recurrence using various types of examination images is also a feasible approach, but the relative performance is not particularly impressive. In contrast, several other studies have shown that models combining radiomics data with common clinical features exhibit predictive performance with better results [31,32,34].

#### Quality Assessment

**Figure 3:**
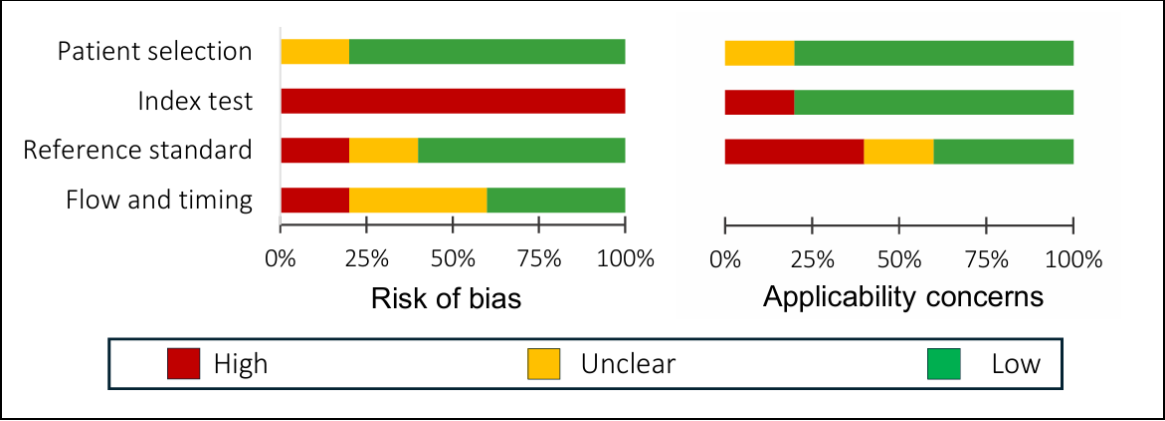
Summary of the assessment results of the Quality Assessment of Diagnostic Accuracy Studies-2 (QUADAS-2) tool on 5 patient classification and image segmentation studies in subgroup A.

The risk of bias and applicability of the included studies in subgroup A (5/23, 22%; targeting patients classification and image segmentation) were confirmed by using the adapted QUADAS-2 tool. The results presented in Figure 3 indicate that the “index test” domain is the most susceptible to bias. All studies exhibit a high risk of bias in this domain because they rely solely on single-center data for model development and lack external validation, which is essential to fully assess the model’s generalizability. The applicability of 60% (3/5) of the studies showed unclear or high risk of bias in different fields.

**Figure 4:**
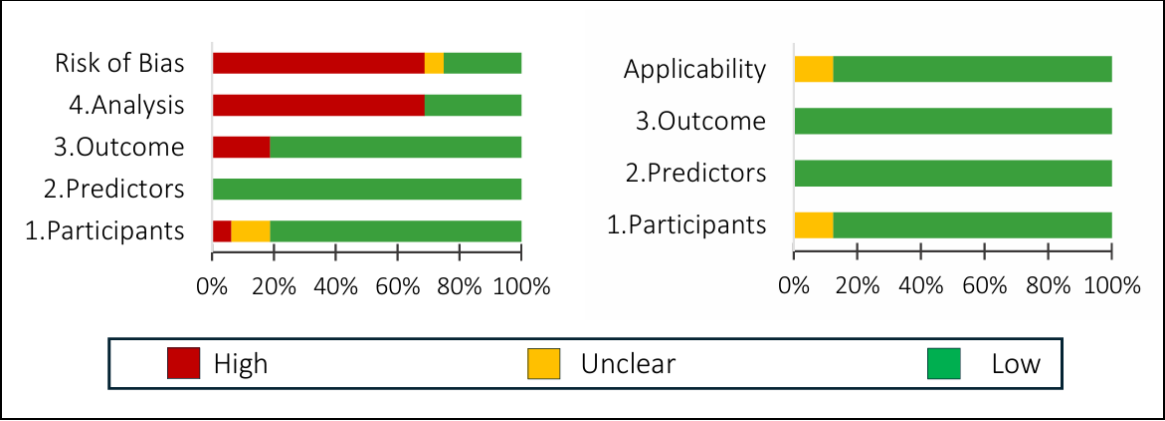
Summary of the assessment results of the Prediction model Risk Of Bias Assessment Tool (PROBAST) on 16 patient prognosis prediction studies in subgroup B.

For subgroup B (16/23, 70%; targeting patient prognosis prediction), the result indicated that the overall risk of bias and applicability was determined to be low in 25% (4/16) of the studies and high in 69% (11/16) of the studies, and the latter was also due to insufficient consideration of model generalization issues in the assessment of the “analysis” domain, resulting in a high risk of bias. Furthermore, 12% (2/16) of the studies were rated as uncertain risk in terms of applicability due to unclear inclusion criteria for the study participants. The results are shown in Figure 4.

The detailed risk of bias and applicability assessment results for both subgroups are shown in Figure S1 and Table S5 in Multimedia Appendix 1.

## Discussion

### Principal Findings

ML has shown great advantages in processing and analyzing medical data and has been increasingly applied to solve various medical problems [24], including those in the field of AF. Compared with several existing ML-related reviews in this field [55-57], we focus more on the research of applying ML in the management of patients undergoing AFCA. We systematically evaluated the 23 highly relevant studies and found that ML performed well overall in AFCA patient management. Models constructed in several studies performed as well as human clinicians in identifying potential ablation targets (rotors and focal sources) and assisting in improving ablation strategies (left atrium scar segmentation) [48,49,53]. It should be noted that comparisons between all included studies are hampered to some extent by the heterogeneity of ML results, and some findings may require discussion at a smaller granularity.

### General Overview of the Studies

The results of our review suggest that the application of ML techniques to the prognosis of patients undergoing AFCA seems promising and has received the most attention from researchers within the scope of this review (16/23, 70%). Compared with traditional prediction models, ML models have a better prediction effect on the long-term recurrence of AF after CA. In the past decade, the construction of AF recurrence prediction models was mainly based on 2 methods; the first is to directly use clinical risk scores, such as APPLE [19], CAAP-AF [58], and MB-LATER [59]. The second is to use some specific biomarkers of patients with AF (such as LA volume [60] and LA sphericity index [61]) or to combine the scoring system with clinical variables and biomarkers for statistical inference. Several different evaluation studies have shown that the AUC range for the prediction of AF recurrence of the models constructed using the abovementioned methods is 0.55 to 0.67 [19,20,22], which is lower than the results of ML models reported in most of the included studies. Most of these ML models achieved performance values >0.8 in terms of AUC, accuracy, and *F*_1_-score indicators. The reason for the poor predictive performance of the traditional methods may lie in the limitations of the predictor dimensions, as scoring and statistical models usually rely on previous literature to select a limited number of clinical variables and specific markers due to the need to fully consider clinical ease of use and interpretability, which also leads to some extent to the neglect of the interactions between variables.

In contrast, ML models are capable of handling much larger and diverse datasets, particularly including imaging and electrophysiologic signal data that were not directly amenable to statistical analysis in the past. Through comprehensive learning of complex data patterns and nonlinear relationships, ML models can achieve more accurate predictive results, which will have many positive impacts for effective intervention in the long-term health of patients undergoing AFCA. On the one hand, the predictive results provided by ML models will guide postprocedure medical management and expectations [37] and help clinicians to take interventions in advance, including medication, lifestyle modifications, or regular follow-ups; on the other hand, the application of ML models can also improve patient engagement in health management and treatment adherence, as in the case of the web calculator developed by Saglietto et al [44], which allows the free personalized prediction of the probability of recurrence after 1 year of AFCA treatment, serving as a decision-support tool that facilitates patient-centered care and joint decision-making between physicians and patients.

Similarly, optimizing CA strategies and identifying potential ablation targets could also benefit from the integration of ML. Because of the different mechanisms of AF triggering and maintenance among patients and the difficulty of identifying patients with one or more of several different mechanisms [16], physicians need to develop ablation strategies and identify ablation targets with the help of different means, such as imaging (including MRI and CT) or invasive electrophysiologic examinations. When dealing with some specific patient groups, such as patients with non–PV-triggered AF, this process not only tests the physician’s clinical experience but also raises the patient’s exposure time to radiation [62]. Several studies in this review demonstrated that the DL models can identify potential ablation targets based on the patient’s EGM data, with 82.5% and 80.4% accuracy[47,49], or guide CA according to personalized appropriate strategies based on LGE-MRI images [51,52], which is conducive to guiding clinicians to implement targeted treatment and shorten the duration of radiation exposure. In addition, using ML technology and patient preoperative data to determine whether additional ablation is needed on the basis of PV isolation is also meaningful for improving patient selection and reducing the risk of postoperative recurrence in patients [31,50]. With the help of ML technology, physicians were given the opportunity to intuitively gain insights from these data into the mechanisms that initiate or maintain arrhythmias in patients, thereby efficiently locating ablation targets, reducing uncertainty in CA treatment and late recurrence, optimizing the allocation of medical resources, and ultimately improving patients’ clinical outcomes.

In terms of model training, using multimodal data from patients with AF to develop ML models might be a promising approach to improve model performance. Models benefit from multimodal data that more fully reflect the demographic characteristics, clinical features, and actual cardiac condition with electrophysiological characteristics of patients with AF, as reported in some previous studies [31,32,34,37]. In traditional prognostic studies involving patients who have undergone ablation, factors such as AF type, ejection fraction values, LA volume [63], and obstructive sleep apnea [10] have been acknowledged as potential predictors that may be independently or jointly associated with recurrence. However, after comprehensively analyzing the cardiac morphological structure, electrophysiological structure, and clinical characteristics with the help of ML, factors that have been ignored, such as fractal characteristics [31,32], have attracted attention. This suggests that ML will reveal previously unknown associations and patterns that will drive new insights and innovations in the management of patients undergoing CA for AF. It is worth noting that the use of ML techniques to process multimodal data is common across the medical field (eg, oncology [64] and lymphocytosis [65]), but it needs to be further popularized in the field of AF, particularly for the management of patients undergoing AFCA.

In addition, compared with classical ML methods, DL seems to have more obvious advantages in the field of AFCA patient management. As shown in Tables 1 and 4, >60% (14/23, 61%) of the studies included in this scoping review used DL technology, and a notable characteristic of this subset of studies is that many involved data modalities beyond general clinical features, encompassing imaging data (CT, echocardiography, etc) or electrophysiological signal (ECG, EGM, etc). This is attributed to DL’s demonstrated prowess in complex feature learning, time series processing, and other aspects, contributing to its gradual emergence as a mainstream technology in the field of computer-aided detection and diagnosis [66]. In a blinded, randomized controlled trial designed by He et al [67] for DL algorithm, the initial assessment of LV ejection fraction by DL was even superior to that of sonographers. Therefore, we believe that in the future, there will be more data-driven instances of applying DL to AFCA patient management to promote substantive progress in this field, such as optimizing treatment strategies, reducing the risk of complications, and conducting early intervention in patient populations prone to recurrence.

### Existing Limitations of the ML Models

We also noted some general limitations to the current use of ML techniques in AFCA patient management, which have hindered its more comprehensive implementation. In using the adapted QUADAS-2 and PROBAST to assess the risk of bias for all included studies, we found that most of the studies (19/23, 83%) were conducted based on single-center, retrospective data and lacked external validation, which raises concerns as to whether ML models can be generalized to a high level. Limited by data availability, some ML models with excellent results will not be perfectly reproduced on other datasets. In addition, the absence of an external validation set may result in overestimating the model’s performance, preventing an accurate assessment of the ML model’s true capability [68]. Therefore, how to break through the limitations of single-center studies is an issue that has to be considered when further promoting the in-depth use of ML.

In addition, there remain some difficulties in effectively comparing and evaluating the results of ML models applied to the management of patients undergoing AFCA. We found that even for the similar medical task (eg, patient prognosis prediction or ablation strategy improvement), existing studies still showed significant heterogeneity in the reporting and analysis of results [36,46], which hinders objective comparisons between the results of different ML models.

It is also worth noting that when considering the use and introduction of ML techniques from a practical perspective, we should correctly recognize that there are differences in the level of trust and acceptance of ML among different individuals (both physicians and patients). This phenomenon is partly caused by the “black box” problem of some DL models [69], which was rarely discussed in the studies included in this scoping review. Without the ability to understand how models make decisions, physicians will be more likely to trust their own clinical experience and medical knowledge to confirm treatment decisions based on interviews, symptom judgments, and clinical examinations, as they may not be willing to take responsibility for suboptimal results of ML models [70]. More critically, the “black box” prevents clinicians and researchers from fully understanding or explaining the principles behind model decisions [55], which is a limitation that cannot be ignored for research with the original intention of explaining medical problems.

### Future Research

In future studies, the composition of the patient cohort and the construction of the dataset are primary issues that need to be considered. The challenges of conducting multicenter studies primarily stem from the legal, ethical, and privacy constraints associated with sharing raw AFCA patient data [71]. Distributed learning, represented by paradigms such as integrated learning and federated learning, would be a strategy for future studies to realize effective collaboration among multicenters [72]. In a network of distributed learning nodes, individual institutions do not need to share patient data externally. Instead, they contribute to constructing the final model across multiple centers by updating the model (eg, adjusting the weighting parameters) [73].

In addition, efforts should be accelerated to develop standardized protocols and consensus guidelines for the evaluation of ML models, thus helping clinicians in selecting more appropriate ML techniques to address specific clinical tasks in the management of patients undergoing AFCA [74,75].

To address the issue of limited clinical interpretability of ML models, commonly used techniques in medical research, such as Shapley Additive Explanation and attention mechanisms [45,76-78], can generate input importance maps to highlight the features that contribute most to model detection. We believe that more research will be devoted to opening this “black box” in the future [79]. Whether it is physicians, patients, or researchers, the real hope is that ML will be able to prompt as much human-understandable auxiliary information as possible to prove its usefulness as an additional decision point in the complex, changing, practice-based patient management environment of AFCA.

### Strengths and Limitations

To the best of the authors’ knowledge, this is the first scoping review of studies applying ML techniques to the management of patients undergoing AFCA. We summarized the various ML methods, variables, model results, and clinical application scenarios that have emerged; emphasized the huge potential for further in-depth application of ML and the limitations that need to be overcome; and provided guidance for future research in terms of data preparation and model selection. Moreover, some sources of evidence were critically evaluated using modified QUADAS-2 and PROBAST to make the conclusions of the review more reliable and convincing. This study has several limitations. First, the results of the included studies could not be synthesized into quantitative analysis. Although all studies reported model performance, different indicators were not directly comparable. Second, this review only focused on studies published in English and did not cover other languages and gray literature, which may have led to the omission of some important findings in this field.

### Conclusions

The rapid development of ML has injected vitality into the transformation of AFCA patient management to become more precise and reliable. It could improve the identification of ablation targets, ablation strategies, and prediction of patient prognosis, which will effectively improve treatment effectiveness and patient management efficiency. Despite its broad prospects, the widespread and in-depth application of ML technology in AFCA patient management still requires consideration of issues from data and the model itself, such as data quality, model generalization, and model interpretability.

## Appendix

Multimedia Appendix 1 Supplementary tables and figures.

Multimedia Appendix 2 PRISMA (Preferred Reporting Items for Systematic Reviews and Meta-Analyses) checklist.

## Abbreviations

AF: atrial fibrillation
AFCA: atrial fibrillation catheter ablation
AUC: area under the receiver operating characteristic curve
CA: catheter ablation
CNN: convolutional neural network
CT: computed tomography
DL: deep learning
ECG: electrocardiogram
EGM: electrogram
EHR: electronic health record
LGE-MRI: late gadolinium-enhanced cardiac magnetic resonance
LV: left ventricular
ML: machine learning
PRISMA-ScR: Preferred Reporting Items for Systematic Reviews and Meta-Analyses extension for Scoping Reviews
PROBAST: Prediction model Risk Of Bias Assessment Tool
PV: pulmonary vein
QUADAS-2: Quality Assessment of Diagnostic Accuracy Studies-2

## Data Availability

All data generated or analyzed during this study are included in this published article and Multimedia Appendices 1 and 2.
