# Supplementary material for: Machine Learning in the Management of Patients Undergoing Catheter Ablation for Atrial Fibrillation: Scoping Review
Source: J Med Internet Res. 2025 Feb 10;27:e60888. doi: 10.2196/60888 (PMC11851043; doi:10.2196/60888)
Supplement: Multimedia Appendix 1 [file jmir_v27i1e60888_app1.docx]

**Supplementary Table 1. Search strategies and results**

| **Database** | **Retrieval strategy** | **Results** |
| --- | --- | --- |
| **PubMed** | ("Atrial Fibrillation"[Mesh] OR atrial fibrillation[tiab] OR Afib[tiab]) AND ("Catheter Ablation"[Mesh] OR catheter ablation[tiab]) AND (machine learning or deep learning or artificial intelligence) NOT REVIEW | 136 |
| **Web of Science** | ((TS=(atrial fibrillation) OR TS=(afib) OR TS=(AF)) AND TS=(catheter ablation)) AND (TS=(machine learning) OR TS=(deep learning) OR TS=(artificial intelligence)) and Preprint Citation Index (Exclude – Database) and Other or Review Article or Meeting or Editorial Material or Early Access or Dissertation Thesis or Abstract or Unspecified or Letter or Patent or Correction (Exclude – Document Types) | 57 |
| **Cochrane** | (MeSH descriptor: [Atrial Fibrillation] explode all trees) AND (MeSH descriptor: [Catheter Ablation] explode all trees) AND (((machine learning) OR ((deep learning) OR (artificial intelligence)):ti, ab, kw) AND (((machine learning) OR ((deep learning) OR (artificial intelligence)):ti, ab, kw)). deep learning) OR (artificial intelligence)):ti, ab, kw) | 4 |
| **Embase** | ('atrial fibrillation':ti,ab,kw OR af:ti,ab,kw OR afib:ti,ab,kw) AND 'catheter ablation':ti,ab,kw AND ('machine learning':ti,ab,kw OR 'deep learning':ti, ab, kw OR 'artificial intelligence':ti, ab, kw) AND [article]/lim | 34 |
| **ScienceDirect** | ((atrial fibrillation) OR (af) OR (afib)) and (catheter ablation) and ((deep learning) OR (machine learning) OR (artificial intelligence)) | 12 |

**Supplementary Table 2. Adapted QUADAS-2 used assessment questions for risk of bias and applicability assessment**

| **Domain** | **Signal questions** |
| --- | --- |
| **Patient Choice** | 1. Were data imbalances (if there were any) addressed in the study?  2. Did the study avoid inappropriate data exclusion?  3. Was a consecutive or random sample of patients (or data) enrolled?  4. Was the test dataset separate from the training and validation datasets?  **Applicability:** Are there concerns about the inclusion of data and settings that are not consistent with the synthesized question? |
| **Index Test** | 1. Were the results of deep learning methods interpreted without knowledge of the results of the reference standard?  2. If a threshold was used, was it pre-specified?  3. Was the methodology described in sufficient detail to reproduce the results presented?  4. Was external verification performed (or was multi center data being uesed)?  5. Did the study conduct robustness or sensitivity analyses of its models?  **Applicability:** Are there problems with methods, behaviors, or interpretations that differ from the synthesized problem? |
| **Reference Standard** | 1. Were the reference standard results interpreted without knowledge of the results of the indicator tests?  2. Did the study use the gold standard?  3. If not, was the annotation procedure described in the study and found to minimize bias?  4. Did the study adequately report its limitations, biases, or generalizability issues?  **Applicability:** Are there problems where the target conditions defined by the reference standard do not correspond to the problem? |
| **Process & Timing** | 1. Were all data included in the analysis?  2. Were there appropriate intervals between indicator testing and reference standards?  3. Had all patients undergone the gold standard test?  4. Were there accurate reference standards for all data? |

**Supplementary Table 3. PROBAST used assessment questions for risk of bias and applicability assessment**

| **Domain** | **Signal questions** |
| --- | --- |
| **Participants** | 1.1 Were appropriate data sources used, e.g. cohort, RCT or nested case-control study data?  1.2 Were all inclusions and exclusions of participants appropriate?  **ROB:** Risk of bias introduced by selection of participants. (low/ high/ unclear)  **Applicability:** Concern that the included participants and setting do not match the review question. (low/ high/ unclear) |
| **Predictors** | 2.1 Were predictors defined and assessed in a similar way for all participants?  2.2 Were predictor assessments made without knowledge of outcome data?  2.3 Are all predictors available at the time the model is intended to be used?  **ROB:** Risk of bias introduced by predictors or their assessment. (low/ high/ unclear)  **Applicability:** Concern that the definition, assessment or timing of predictors in the model do not match the review question. (low /high /unclear) |
| **Outcome** | 3.1 Was the outcome determined appropriately?  3.2 Was a pre-specified or standard outcome definition used?  3.3 Were predictors excluded from the outcome definition?  3.4 Was the outcome defined and determined in a similar way for all participants?\  3.5 Was the outcome determined without knowledge of predictor information?  3.6 Was the time interval between predictor assessment and outcome determination appropriate?  **ROB:** Risk of bias introduced by the outcome or its determination. (low /high /unclear)  **Applicability:** Concern that the outcome, its definition, timing or determination do not match the review question. (low /high /unclear) |
| **Analysis** | 4.1 Were there a reasonable number of participants with the outcome? i.e. Was the sample size adequately powered?  4.2 Were continuous and categorical predictors handled appropriately?  4.3 Were all enrolled participants included in the analysis?  4.4 Were participants with missing data handled appropriately?  4.5 Was selection of predictors based on univariable analysis avoided?  4.6 Were complexities in the data accounted for appropriately?  4.7 Were relevant model performance measures evaluated appropriately?  4.8 Were model overfitting and optimism in model performance accounted for?  4.9 Do predictors and their assigned weights in the final model correspond to the results from the reported multivariable analysis?  **ROB:** Risk of bias introduced by the analysis. (low /high /unclear) |

**Supplementary Table 4. List of studies excluded during the full-text screening stage and reasons for exclusion**

| **Author, year** | **Title** | **Reasons for exclusion** |
| --- | --- | --- |
| Miguel, et al.,2021 | Non-invasive Estimation of Atrial Fibrillation Driver Position With Convolutional Neural Networks and Body Surface Potentials | The objectives of the study design deviate from the review topic |
| Baalman, et al., 2021 | Prediction of Atrial Fibrillation Recurrence after Thoracoscopic Surgical Ablation Using Machine Learning Techniques | Focus on thoracoscopic surgical ablation rather than catheter ablation |
| Zolotarev, et al., 2022 | Optical Mapping-Validated Machine Learning Improves Atrial Fibrillation Driver Detection by Multi-Electrode Mapping | Studying on ex-vivo human hearts, not patients with atrial fibrillation |
| Jorge, et al., 2021 | Using Machine Learning to Characterize Atrial Fibrotic Substrate From Intracardiac Signals With a Hybrid in silico and in vivo Dataset | Using simulated data rather than real patients |
| Chen, et al., 2020 | Automated extraction of left atrial volumes from two-dimensional computer tomography images using a deep learning technique | The objectives of the study design deviate from the review topic |
| Musa, et al.,2022 | Generalizable Framework for Atrial Volume Estimation for Cardiac CT Images Using Deep Learning With Quality Control Assessment | The objectives of the study design deviate from the review topic |
| Seno, et al., 2021 | In-Silico Deep Reinforcement Learning for Effective Cardiac Ablation Strategy | Using simulated data rather than real patients |
| Lee, et al., 2021 | Left Atrial Wall Stress and the Long-Term Outcome of Catheter Ablation of Atrial Fibrillation: An Artificial Intelligence-Based Prediction of Atrial Wall Stress | The objectives of the study design deviate from the review topic |
| Zheng, et al, 2023 | Identification of Recurrent Atrial Fibrillation using Natural Language Processing Applied to Electronic Health Records | The usage method is not a machine learning method |
| Lee, et al, 2021 | Left Atrial Wall Stress and the Long-Term Outcome of Catheter Ablation of Atrial Fibrillation: An Artificial Intelligence-Based Prediction of Atrial Wall Stress | The objectives of the study design deviate from the review topic |
| Barrett, et al.,2023 | Evaluation of Quantitative Decision-Making for Rhythm Management of Atrial Fibrillation Using Tabular Q-Learning | The objectives of the study design deviate from the review topic |

**Supplementary Table 5. Details of the assessment results of the PROBAST for 16 studies in subgroup B**

| **Author, Year** | **Risk of Bias** | | | | **Applicability** | | | **Overall** | |
| --- | --- | --- | --- | --- | --- | --- | --- | --- | --- |
|  | 1. Participants | 2. Predictors | 3. Outcome | 4. Analysis | 1. Participants | 2. Predictors | 3. Outcome | **Risk of Bias** | **Applicability** |
| Firouznia, 2021 | + | + | + | - | + | + | + | - | + |
| Atta-Fosu, 2021 | + | + | + | - | + | + | + | - | + |
| Shade, 2020 | + | + | - | - | + | + | + | - | + |
| Roney, 2022 | + | + | + | - | + | + | + | - | + |
| Hwang, 2021 | + | + | - | - | + | + | + | - | + |
| An, 2022 | - | + | + | - | ？ | + | + | - | ？ |
| Tang, 2022 | + | + | + | - | + | + | + | - | + |
| Li, 2019 | ? | + | - | - | ？ | + | + | - | ? |
| Saiz-Vivo, 2021 | + | + | + | + | + | + | + | + | + |
| Jiang, 2023 | + | + | + | - | + | + | + | - | + |
| Lee, 2022 | + | + | + | + | + | + | + | + | + |
| Park, 2022 | + | + | + | + | + | + | + | + | + |
| Hung, 2020 | ? | + | + | + | + | + | + | ? | + |
| Saglietto, 2023 | + | + | + | + | + | + | + | + | + |
| Ma, 2023 | + | + | + | - | + | + | + | - | + |
| Zhou, 2022 | + | + | + | - | + | + | + | - | + |

ROB = risk of bias; “+” indicates low ROB/low concern regarding applicability; “-" indicates high ROB/high concern regarding applicability; and ? indicates unclear ROB/unclear concern regarding applicability.

**About overall ROB:**

(1)Low risk of bias: If all domains were rated low risk of bias.

(2) High risk of bias: If at least one domain is judged to be at high risk of bias.

(3) Unclear risk of bias: If an unclear risk of bias was noted in at least one domain and it was low risk for all other domains.

**About overall applicability:**

(1) Low concerns regarding applicability: If low concerns regarding applicability for all domains, the prediction model evaluation is judged to have low concerns regarding applicability.

(2) High concerns regarding applicability: If high concerns regarding applicability for at least one domain, the prediction model evaluation is judged to have high concerns regarding applicability.

(3) Unclear concerns regarding applicability: If unclear concerns (but no “high concern”) regarding applicability for at least one domain, the prediction model evaluation is judged to have unclear concerns regarding applicability overall.

**Supplementary Table 6. Complete ML model information and performance of the included studies.**

| **Author, year**  **(Reference)** | **Medical missions** | **Algorithms/Models** | **Model Performance** | | | | | **External validation** |
| --- | --- | --- | --- | --- | --- | --- | --- | --- |
|  |  |  | **AUC** | **SE** | **SP** | **ACC** | **Other** |  |
| Liu, et al.^1^ | 3 | ResNet34 | 0.88 | 75.0 | 95.7 | 88.6 | NR | No |
| Firouznia, et al. ^2^ | 1 | RF | 0.87 | NR | NR | NR | NR | No |
| Atta-Fosu, et al. ^3^ | 1 | XGBoost | 0.78 | NR | NR | NR | NR | Yes |
| Muizniece, et al. ^4^ | 3 | Reinforcement Q learning algorithm | NR | NR | NR | 72% | NR | No |
| Muffoletto, et al. ^5^ | 3 | CNN | NR | NR | NR | 78.68-86.50% | NR | No |
| Shade, et al. ^6^ | 1 | RF, QDA* | 0.82 | 82% | 89% | NR | NR | No |
| Roney, et al. ^7^ | 1 | KNN, SVM, RF, LR, SVM* | 0.85 | NR | NR | NR | F1-Score=0.8  PR=0.74 | No |
| Yang, et al. ^8^ | 3 | MVVT | NR | 91.96 | 99.36 | 98.59 | DI=93.11 | Yes |
| Hwang, et al. ^9^ | 1 | CNN | 0.861 | 0.803 | 0.789 | 0.796 | NR | No |
| Liao, et al. ^10^ | 2 | Improved residual convolutional neural network based on ResNet18*, LR, SVM, KNN | 0.980 | 78.1% | 82.2% | NR | NR | No |
| Ríos-Muñoz, et al. ^11^ | 2 | CRNN, ATI-CNN, SimpleCNN | 0.81 | NR | NR | NR | MCC=0.680 | No |
| Alhusseini, et al. ^12^ | 2 | CNN | NR | 97% | 93% | 95% | NR | No |
| An, et al. ^13^ | 1 | MLP | 0.5 | NR | NR | 87.5% | F1-Score=0.933  PR-AUC=0.938 | No |
| Tang, et al. ^14^ | 1 | CatBoost, CNN* | 0.859 | NR | NR | NR | NR | No |
| Li, et al. ^15^ | 1 | CNN-SVM | NR | 88% | 96% | 96% | NR | No |
| Saiz-Vivo, et al. ^16^ | 1 | SVM, KNN, Regression Trees, OWV* | NR | 76% | 87% | 82% | NR | No |
| Jiang, et al. ^17^ | 1 | CNN | 0.84 | 72.3% | 95% | 92% | PR=0.69  F1-Score=0.71 | No |
| Lee, et al. ^18^ | 1 | XGBoost, LN, SVM, MLP* | 0.766 | NR | NR | NR | NR | No |
| Park, et al. ^19^ | 1 | RF | 0.965 | 96% | 89% | NR | NR | Yes |
| Hung, et al.^20^ | 1 | KNN*, DT, SVM | 0.91 | 71.3% | 99.1% | 85.4% | PR=0.886 | No |
| Saglietto, et al. ^21^ | 1 | DT, RF, AdaBoost, KNN, RF*. | 0.721 | NR | NR | NR | NR | Yes |
| Ma, et al. ^22^ | 1 | RF | 0.667 | NR | NR | NR | NR | No |
| Zhou, et al. ^23^ | 1 | CNNSURV (CNN+COX) | 0.76 | NR | NR | NR | CI=0.76 | No |

**Note:** Interpretation of medical task codes: 1, prognosis for patients with catheter ablation of AF; 2, identification of ablation targetst; 3, ablation strategy improvement. If more than one ML model was used in a given study, the model marked with * had the best performance.

**Abbreviations:** AUC, area under the curve; SE, sensitivity; SP, specificity; ACC, accuracy; PR, precision; PR-AUC, precision-recall curve; DI, dice score; MCC, Matthews correlation coefficient; CI, C-index; PVCT, pulmonary vein computed tomography; LGE-MRI, late gadolinium-enhanced cardiac magnetic resonance; ECG, electrocardiogram; uEGM, unipolar endocardial electrogram; bEGM, bipolar endocardial electrogram; CT, computed tomography; uLAT, unipolar local activation time; BSPM: body surface potential map; CNN: convolutional neural network; CRNN: convolutional recurrent neural network; RF: random forest; QDA: quadratic discriminant analysis; KNN: k-nearest neighbor; SVM: vector machine; MVTT: Fully Automated Multi-View Two-Task Recursive Attention Model; LR: Logistic Regression; DT: Decision Tree; OWV: Optimal Weighted Voting; MLP: Multi-Layer Perceptron; NR: Not Reported.

**Supplementary Figre 1.** **Details of the assessment results of the QUADAS-2 tool for 5 studies in subgroup A**


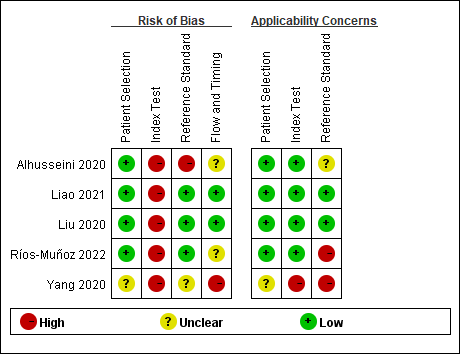


**Referesnces**

1. Liu CM, Chang SL, Chen HH, et al. The Clinical Application of the Deep Learning Technique for Predicting Trigger Origins in Patients With Paroxysmal Atrial Fibrillation With Catheter Ablation. *Circ Arrhythm Electrophysiol*. 2020;13(11):e008518. doi:10.1161/circep.120.008518

2. Firouznia M, Feeny AK, LaBarbera MA, et al. Machine Learning-Derived Fractal Features of Shape and Texture of the Left Atrium and Pulmonary Veins From Cardiac Computed Tomography Scans Are Associated With Risk of Recurrence of Atrial Fibrillation Postablation. *Circ Arrhythm Electrophysiol*. 2021;14(3):e009265. doi:10.1161/circep.120.009265

3. Atta-Fosu T, LaBarbera M, Ghose S, et al. A new machine learning approach for predicting likelihood of recurrence following ablation for atrial fibrillation from CT. *BMC Med Imaging*. 2021;21(1):45. doi:10.1186/s12880-021-00578-4

4. Muizniece L, Bertagnoli A, Qureshi A, et al. Reinforcement Learning to Improve Image-Guidance of Ablation Therapy for Atrial Fibrillation. *Front Physiol*. 2021;12:733139. doi:10.3389/fphys.2021.733139

5. Muffoletto M, Qureshi A, Zeidan A, et al. Toward Patient-Specific Prediction of Ablation Strategies for Atrial Fibrillation Using Deep Learning. *Front Physiol*. 2021;12:674106. doi:10.3389/fphys.2021.674106

6. Shade JK, Ali RL, Basile D, et al. Preprocedure Application of Machine Learning and Mechanistic Simulations Predicts Likelihood of Paroxysmal Atrial Fibrillation Recurrence Following Pulmonary Vein Isolation. *Circ Arrhythm Electrophysiol*. 2020;13(7):e008213. doi:10.1161/circep.119.008213

7. Roney CH, Sim I, Yu J, et al. Predicting Atrial Fibrillation Recurrence by Combining Population Data and Virtual Cohorts of Patient-Specific Left Atrial Models. *Circ Arrhythm Electrophysiol*. 2022;15(2):e010253. doi:10.1161/circep.121.010253

8. Yang G, Chen J, Gao Z, et al. Simultaneous left atrium anatomy and scar segmentations via deep learning in multiview information with attention. *Future Generation Computer Systems-the International Journal of Escience*. 2020;107:215-228. doi:10.1016/j.future.2020.02.005

9. Hwang YT, Lee HL, Lu CH, et al. A Novel Approach for Predicting Atrial Fibrillation Recurrence After Ablation Using Deep Convolutional Neural Networks by Assessing Left Atrial Curved M-Mode Speckle-Tracking Images. *Front Cardiovasc Med*. 2020;7:605642. doi:10.3389/fcvm.2020.605642

10. Liao S, Ragot D, Nayyar S, et al. Deep Learning Classification of Unipolar Electrograms in Human Atrial Fibrillation: Application in Focal Source Mapping. *Front Physiol*. 2021;12:704122. doi:10.3389/fphys.2021.704122

11. Ríos-Muñoz GR, Fernández-Avilés F, Arenal Á. Convolutional Neural Networks for Mechanistic Driver Detection in Atrial Fibrillation. *Int J Mol Sci*. 2022;23(8). doi:10.3390/ijms23084216

12. Alhusseini MI, Abuzaid F, Rogers AJ, et al. Machine Learning to Classify Intracardiac Electrical Patterns During Atrial Fibrillation: Machine Learning of Atrial Fibrillation. *Circ Arrhythm Electrophysiol*. 2020;13(8):e008160. doi:10.1161/CIRCEP.119.008160

13. An Q, McBeth R, Zhou H, et al. Prediction of Type and Recurrence of Atrial Fibrillation after Catheter Ablation via Left Atrial Electroanatomical Voltage Mapping Registration and Multilayer Perceptron Classification: A Retrospective Study. *Sensors (Basel)*. 2022;22(11). doi:10.3390/s22114058

14. Tang S, Razeghi O, Kapoor R, et al. Machine Learning-Enabled Multimodal Fusion of Intra-Atrial and Body Surface Signals in Prediction of Atrial Fibrillation Ablation Outcomes. *Circ Arrhythm Electrophysiol*. 2022;15(8):e010850. doi:10.1161/circep.122.010850

15. Li Z, Feng X, Wu Z, Yang C, Bai B, Yang Q. Classification of Atrial Fibrillation Recurrence Based on a Convolution Neural Network With SVM Architecture. *IEEE Access*. 2019;7:77849-77856. doi:10.1109/ACCESS.2019.2920900

16. Saiz-Vivo J, Corino VDA, Hatala R, de Melis M, Mainardi LT. Heart Rate Variability and Clinical Features as Predictors of Atrial Fibrillation Recurrence After Catheter Ablation: A Pilot Study. *Front Physiol*. 2021;12:672896. doi:10.3389/fphys.2021.672896

17. Jiang J, Deng H, Liao H, et al. An Artificial Intelligence-Enabled ECG Algorithm for Predicting the Risk of Recurrence in Patients with Paroxysmal Atrial Fibrillation after Catheter Ablation. *J Clin Med*. 2023;12(5). doi:10.3390/jcm12051933

18. Lee DI, Park MJ, Choi JW, Park S. Deep Learning Model for Predicting Rhythm Outcomes after Radiofrequency Catheter Ablation in Patients with Atrial Fibrillation. *J Healthc Eng*. 2022;2022:2863495. doi:10.1155/2022/2863495

19. Park JW, Kwon OS, Shim J, et al. Machine Learning-Predicted Progression to Permanent Atrial Fibrillation After Catheter Ablation. *Front Cardiovasc Med*. 2022;9:813914. doi:10.3389/fcvm.2022.813914

20. Hung M, Lauren E, Hon E, et al. Using Machine Learning to Predict 30-Day Hospital Readmissions in Patients with Atrial Fibrillation Undergoing Catheter Ablation. *J Pers Med*. 2020;10(3). doi:10.3390/jpm10030082

21. Saglietto A, Gaita F, Blomstrom-Lundqvist C, et al. AFA-Recur: an ESC EORP AFA-LT registry machine-learning web calculator predicting atrial fibrillation recurrence after ablation. *Europace*. 2023;25(1):92-100. doi:10.1093/europace/euac145

22. Ma Y, Zhang D, Xu J, et al. Explainable machine learning model reveals its decision-making process in identifying patients with paroxysmal atrial fibrillation at high risk for recurrence after catheter ablation. *BMC Cardiovasc Disord*. 2023;23(1):91. doi:10.1186/s12872-023-03087-0

23. Zhou X, Nakamura K, Sahara N, et al. Deep Learning-Based Recurrence Prediction of Atrial Fibrillation After Catheter Ablation. *Circ J*. 2022;86(2):299-308. doi:10.1253/circj.CJ-21-0622
